# Beyond Single-Lead ECG-Derived Respiration Analysis: Use of Vectorcardiograms from the EASI-System for Breathing Frequency Estimation—A Feasibility Study

**DOI:** 10.3390/s26123673

**Published:** 2026-06-09

**Authors:** Felix Maximillian Kuon, Lucas Bohlen, Laura Jacobsen, Markus Riemenschneider, Jürgen Lorenz

**Affiliations:** 1 Faculty of Life Sciences, Hamburg University of Applied Sciences (HAW Hamburg), Ulmenliet 20, 21033 Hamburg, Germany; felixmaximilian.kuon@haw-hamburg.de (F.M.K.); markus.riemenschneider@haw-hamburg.de (M.R.); 2 Research Department, Osteopathie Schule Deutschland, Weidestraße 118c, 22083 Hamburg, Germany; lbohlen@osteopathie-schule.de (L.B.); jacobsen.laura@gmx.de (L.J.); 3 Institute for Clinical Psychology and Psychotherapy, Medical School Hamburg, Am Kaiserkai 1, 20457 Hamburg, Germany; 4 Institute of Interdisciplinary Exercise Science and Sports Medicine, Medical School Hamburg, Am Kaiserkai 1, 20457 Hamburg, Germany

**Keywords:** electrocardiogram-derived respiration (EDR), vectorcardiography (VCG), EASI lead system, heart rate variability (HRV), paced breathing

## Abstract

Precise respiration assessment is crucial for heart rate variability (HRV) interpretation as respiratory components—particularly respiratory sinus arrhythmia (RSA)—provide essential information on vagally mediated regulation. Conventional single-lead electrocardiogram-derived respiration (EDR) methods measure the amplitude modulation of the QRS-waveform caused by respiratory chest movements. This causes a displacement of the electrical heart axis in relation to the ECG lead axis, typically within the 2D frontal plane of the Einthoven electrode montage. Another approach is based on heartbeat acceleration and deceleration during respective inspiration and expiration causing RR interval modulation. However, interval-based methods depend on the complexity of sympathovagal factors that affect RSA. The present feasibility study accounts for the 3D rotational movement of the electrical heart axis during the respiratory cycle and avoids non-respiratory neuromodulatory confounds. The beat-to-beat cardiac rotation was extracted from Frank-XYZ coordinates reconstructed via a four-electrode EASI device. In a pilot study with data from 19 healthy adults performing acoustically paced breathing (6–18 bpm), three surrogates (${\mathrm{RR-Interval}}_{\mathrm{EDR}}$, ${\mathrm{R-Amplitude}}_{\mathrm{EDR}}$, ${\mathrm{Heartmovement}}_{\mathrm{EDR}}$) were compared using a unified Python 3.11.13 pipeline (3D VCG R-peak detection, multivariate Mahalanobis artifact correction, wavelet-based analysis) against a synthetic reference derived from the instructed breathing schedule. The results demonstrated a consistently lower estimation error and higher reference-based signal-to-noise ratio (refSNR), measuring spectral alignment with the paced-breathing trajectory for ${\mathrm{Heartmovement}}_{\mathrm{EDR}}$ and achieving a mean refSNR of 6.01 dB (vs. 4.62 dB for ${\mathrm{RR-Interval}}_{\mathrm{EDR}}$ and 3.20 dB for ${\mathrm{R-Amplitude}}_{\mathrm{EDR}}$) and a mean absolute estimation error of 0.016 Hz (vs. 0.050 Hz and 0.032 Hz, respectively). Notably, ${\mathrm{Heartmovement}}_{\mathrm{EDR}}$ and ${\mathrm{R-Amplitude}}_{\mathrm{EDR}}$ performance slightly improved at higher heart rates, consistent with the interpretation that higher cardiac sampling density benefits spectral resolution for chest movement-based methods, whereas ${\mathrm{RR-Interval}}_{\mathrm{EDR}}$ showed no significant heart rate dependence. Furthermore, ${\mathrm{Heartmovement}}_{\mathrm{EDR}}$ was compared with the EDR results obtained by applying the Kubios-HRV Premium software (version 3.5.0). Kubios-EDR yielded higher precision at elevated breathing frequencies, whereas ${\mathrm{Heartmovement}}_{\mathrm{EDR}}$ outperformed Kubios-EDR at breathing rates below 10 bpm—a range that is particularly relevant for vagally activating slow breathing protocols or treatments. Future work should validate this method using a direct respiration measurement under spontaneous natural breathing conditions.

## 1. Introduction

The analysis of heart rate variability (HRV) has attracted the interest of researchers in a variety of disciplines. Many studies evaluate interbeat intervals and use either pulse plethysmography or electrocardiography (ECG) to test HRV parameters in relation to cognitive or affective states, or as signs of physical or mental disease conditions. One traditional parameter is the ratio of power spectra in the low-frequency (LF) band relative to the high-frequency (HF) band as a marker of the balance between sympathetic and parasympathetic (vagal) tone. However, it is a well-known phenomenon that breathing strongly influences HRV, causing large intraindividual fluctuation of power spectral amplitudes of interbeat intervals by respiratory sinus arrhythmia (RSA). RSA occurs due to inspiration and expiration causing the respective acceleration and deceleration of the heart rate [1,2]. Menuet et al. [3] suggested a terminological clarification of respiratory HRV and that its amplitude in frequency band analysis should not be misinterpreted as a measure of cardiac vagal tone. There are several methods to include measures of respiration in the analysis of HRV, e.g., by chest belts [4] or through ECG-derived respiration analysis [5,6,7].

Conventional electrocardiogram-derived respiration (EDR) estimation methods model breathing either through RR interval modulation or waveform changes in the QRS complex in single leads. RR interval modulation basically considers the above-described phenomenon of RSA, and thus a neuronal interaction of sympathetic and parasympathetic efferent innervation of the sinoatrial node in relation to the breathing cycle, or a combination of both, as implemented in the Kubios-HRV software package [8]. In contrast, waveform-based EDR methods consider the mechanical influence of respiration on the heart’s position and orientation in the thorax, which causes changes in the ECG signal amplitude and morphology. Chest movement-based changes in heart position cause amplitude variations comparable to the scalar product of two vectors, where the angle between the cardiac dipole and lead direction varies along the respiration cycle. When using single-lead ECGs according to the Einthoven triangle, the vector projection is restricted to the frontal plane, which is not optimal for capturing the three-dimensional heart movement. The use of a 3D vectorcardiogram (VCG) allows a more comprehensive capture of the heart’s spatial orientation and movement, providing a more accurate representation of respiration-induced changes in the ECG signal.

RR interval-based EDR methods have clear limitations. The first is that they are based on RSA, which is a complex phenomenon influenced by multiple factors, including autonomic nervous system activity, respiratory patterns, and individual differences regarding age, gender and health status. Furthermore, if the breathing rate is below 0.15 Hz (nine breaths per minute), the RSA signal overlaps with the low-frequency (LF) band of HRV (0.04–0.15 Hz), which is typically associated with a dominant sympathetic activity, rendering the EDR interval methods vulnerable to misinterpretation of the LF/HF ratio as a measure of sympathovagal balance [9].

A few papers have considered the estimation of EDR from the three-dimensional heart movement using 12-channel ECG recordings [10,11]. They applied inverse transformations—most commonly the inverse Dower transform or the Kors regression method—to reconstruct the Frank VCG from the 12-lead ECG, and then used loop alignment techniques to extract respiratory modulation from the resulting cardiac rotation signal. However, the accuracy of such derived VCGs depends critically on the chosen transformation method and degrades substantially in pathological recordings, where the transformation error can obscure respiratory modulation [12]. Furthermore, 12-lead ECGs are impractical for mobile or ambulatory applications, requiring ten electrodes and a clinical acquisition setup.

By contrast, the four-electrode EASI system can directly reconstruct the 3D Frank vectorcardiogram via a two-step matrix transformation (Section 2.4) without relying on inverse approximations from a redundant lead set. To the best of our knowledge, no study has yet exploited this direct EASI-to-Frank pathway for 3D heart movement-based respiration estimation. To address this gap, we developed a novel approach using a compact mobile ECG device (CardioSecur^®^) that records the four-electrode EASI system via an iOS application.

The R-peak spatial orientation is directly converted into a rotation-based 3D-EDR signal (${\mathrm{Heartmovement}}_{\mathrm{EDR}}$) that tracks the angular displacement of the cardiac dipole independently of its magnitude. To enable direct comparison with established one-dimensional EDR surrogates, the 3D angular information is reduced to a scalar beat-to-beat time series via the Euclidean norm of the two orthogonal rotation angles (Section 2.6). This approach achieves a reduction in dimensionality without loss of spatial information and inherently avoids non-respiratory autonomic confounds.

To validate the advantages of this 3D approach over a range of different breathing rates (6–18 breaths per minute), a pilot study was conducted with data from 19 healthy adults following an acoustically paced breathing protocol (see Section 2.1).

## 2. Methods

### 2.1. Study Population and Breathing Protocol

The analysis is based on ECG recordings from a previously conducted study investigating the retest reliability of RSA in healthy adults (study registration: https://drks.de/search/de/trial/DRKS00034187 (accessed on 2 June 2026); ethics approval protocol number 024-02).

Of the 56 test participants, data from 21 were available in the unprocessed EASI format from CardioSecur^®^ and were therefore suitable for the proposed 3D-EDR analysis. The included sample (N=19 after quality screening, see Section 3.1) comprised 12 males (63.2%) and 7 females (36.8%), with a midpoint age of 33.4 ± 7.5 years (age groups: 10.5% 18–25 years, 57.9% 26–35 years, 26.3% 36–45 years, 5.3% 46+ years).

Each participant contributed a single recording session comprising seven paced-breathing segments of 60 s duration (6–18 bpm), processed independently for all three EDR methods (maximum 19×7×3=399 method–segment pairs). A final dataset of N=380 segments remained after excluding 19 segments due to severe artifacts or R-peak detection failure (14 affecting ${\mathrm{R-Amplitude}}_{\mathrm{EDR}}$, 4 affecting ${\mathrm{RR-Interval}}_{\mathrm{EDR}}$, 1 affecting ${\mathrm{Heartmovement}}_{\mathrm{EDR}}$).

### 2.2. Signal Acquisition

**Figure 1:**
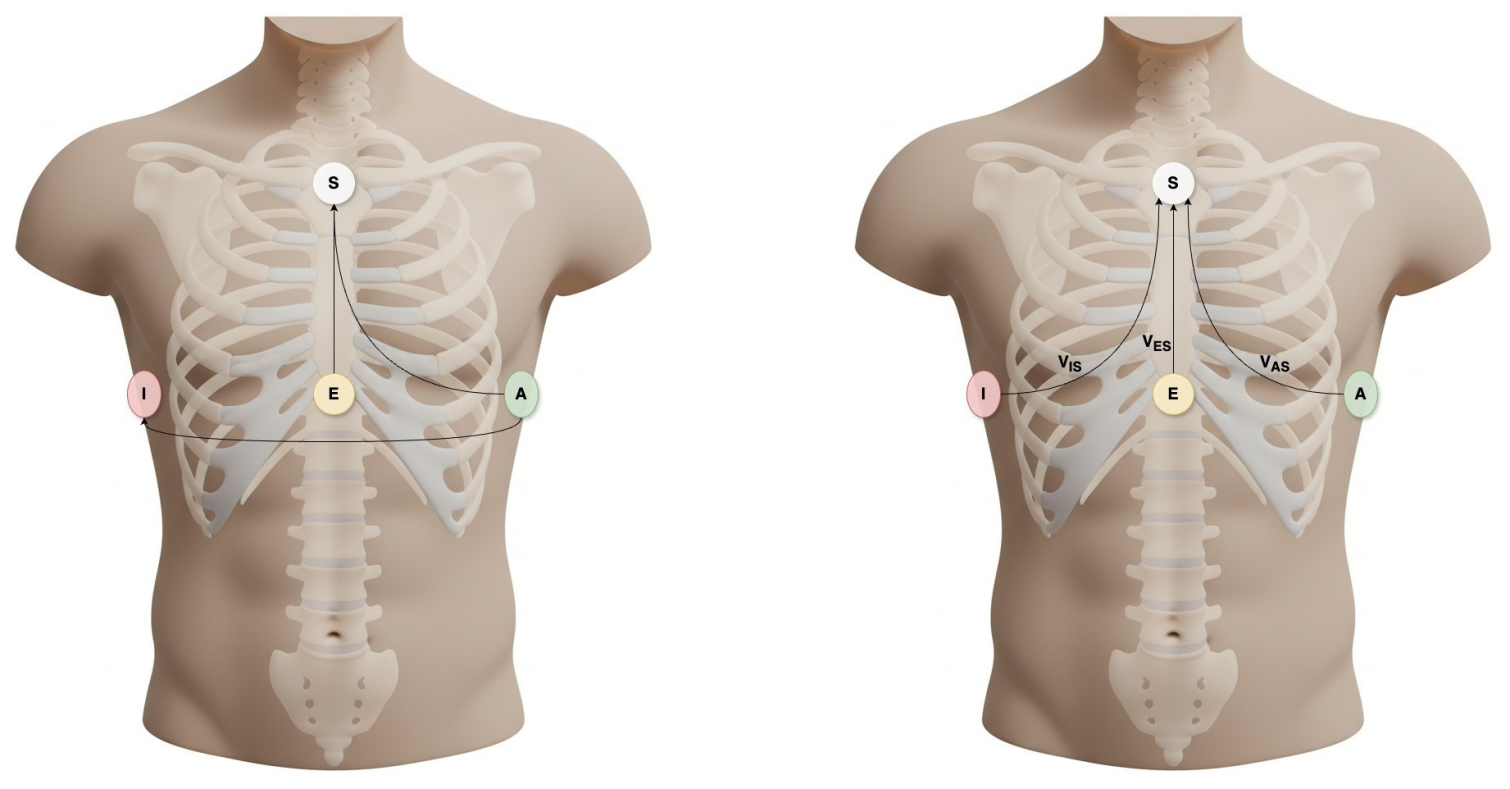
Electrode positions of the EASI system on the thorax. (**left**) Standard EASI electrode positions (E, A, S, I); (**right**) bipolar EASI leads ($V_{IS}$, $V_{ES}$, $V_{AS}$) obtained unprocessed from CardioSecur^®^ raw recordings.

Electrocardiographic data was recorded using a mobile CardioSecur^®^ ECG device based on the EASI electrode scheme [13], which enables multi-lead ECG acquisition using only four surface electrodes [14]. Electrodes were placed on the thorax according to the standard positions E, A, S, and I (Figure 1), yielding the following three bipolar voltage signals from the raw recordings:

- $V_{IS}$: Difference between the right midaxillary line (I) and above the manubrium of the sternum (S) electrode;
- $V_{ES}$: Difference between below the xiphoid process of the sternum (E) and above the manubrium of the sternum (S) electrode;
- $V_{AS}$: Difference between the left midaxillary line (A) and above the manubrium of the sternum (S) electrode.

This reduced electrode setup uses only four surface electrodes (including ground) instead of 10–13 for standard 12-lead ECG, enabling practical mobile data acquisition.

The electrode geometry—with E, A, and I coinciding with the Frank electrode positions [15]—permits subsequent reconstruction of the Frank-XYZ vectorcardiogram [16,17]. This orthogonal representation of the cardiac dipole is well established for lead synthesis and clinical VCG analysis [18].

### 2.3. Preprocessing and Filtering of EASI Signals

Prior to transformation into the Frank-XYZ system, the three EASI signals $V_{IS}$, $V_{ES}$, and $V_{AS}$ were digitized with a sampling frequency of 250 Hz and were bandpass-filtered to suppress low-frequency drift and high-frequency noise without distorting QRS morphology. A linear-phase Finite Impulse Response (FIR) high-pass filter with a cutoff frequency of 1.5 Hz (filter length 201 taps) was applied first, followed by an FIR low-pass filter with a cutoff frequency of 37 Hz (filter length 25 taps). The selected cutoff frequencies restrict the spectrum to the range relevant for QRS detection and HRV analysis, attenuate very slow baseline wander, and remove high-frequency noise as well as myoelectric artifacts [9,19].

### 2.4. Transformation into Frank-XYZ Coordinates

**Figure 2:**
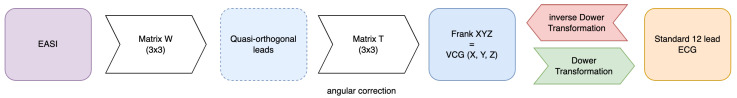
Signal processing pipeline for EASI-to-Frank-XYZ transformation. Starting from four EASI electrodes, intermediate leads are formed and transformed via weighting matrix *W* into quasi-orthogonal coordinates, followed by a second transformation matrix *T* to obtain the Frank coordinate system. The resulting VCG can optionally be converted to a standard 12-lead ECG via the Dower transformation, or inversely derived from a 12-lead ECG via the inverse Dower transformation.

To ensure full methodological transparency and a deterministic, reproducible processing path, the present study implements an explicit reconstruction pipeline from raw EASI potentials to the Frank XYZ coordinate system. The processing chain is illustrated in Figure 2.

First, the raw bipolar lead voltages $V_{IS}$, $V_{ES}$, and $V_{AS}$ (recorded directly as electrode potential differences I–S, E–S, and A–S) are combined into intermediate leads as follows:

$$\mathrm{EI}=V_{ES}-V_{IS},\;\mathrm{AI}=V_{AS}-V_{IS},\;\mathrm{SI}=-V_{IS}.$$

These leads are subsequently mapped into the Frank coordinate system via a two-step linear transformation, following the procedure described in a patent [20]. The first step utilizes the weighting matrix *W* to project the signals into a quasi-orthogonal intermediate space ${\left(X^{\prime },Y^{\prime },Z^{\prime }\right)}^{⊤}$,

$$\left(\begin{array}{c} X^{\prime }\\ Y^{\prime }\\ Z^{\prime } \end{array}\right)=W\left(\begin{array}{c} \mathrm{AI}\\ \mathrm{EI}\\ \mathrm{SI} \end{array}\right),\;W=\left(\begin{array}{ccc} 0.610 & 0.171 & 0.000\\ 0.354 & 0.000 & -1.000\\ 0.869 & -0.605 & 0.000 \end{array}\right).$$

which is then rotated and scaled into the final Frank leads ${\left(X,Y,Z\right)}^{⊤}$ using matrix *T*.

$$\left(\begin{array}{c} X\\ Y\\ Z \end{array}\right)=T\left(\begin{array}{c} X^{\prime }\\ Y^{\prime }\\ Z^{\prime } \end{array}\right),\;T=\left(\begin{array}{ccc} 1.118 & 0.000 & 0.109\\ -0.051 & 0.933 & -0.087\\ -1.108 & 0.000 & 0.772 \end{array}\right).$$

**Figure 3:**
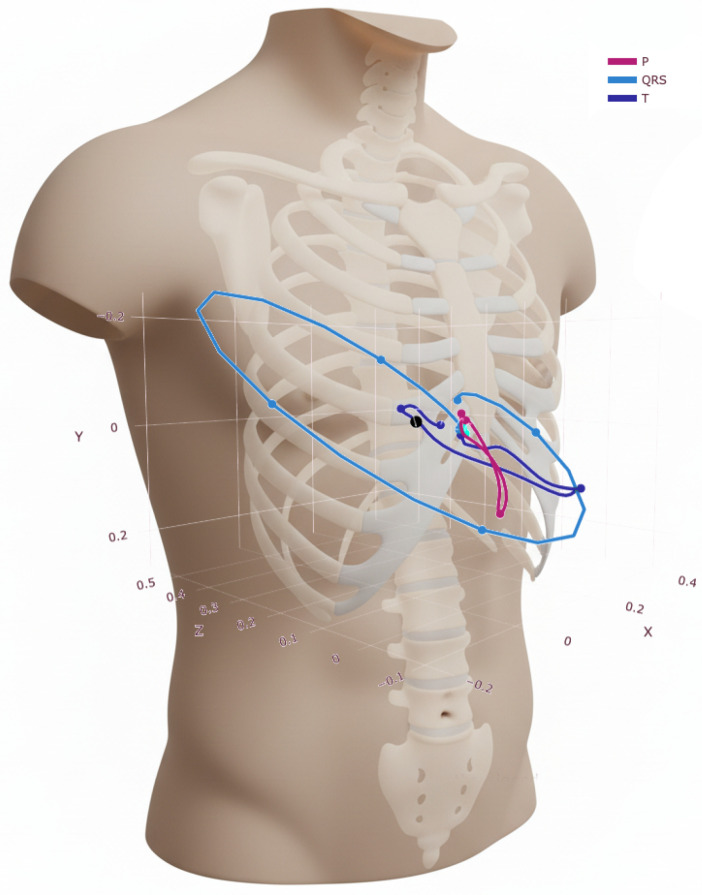
3D vectorcardiogram representation of the cardiac dipole in the Frank coordinate system projected onto the human thorax. P-wave in pink; QRS in blue; T-wave in purple.

The resulting Frank-XYZ coordinates represent an orthogonal vectorcardiographic system, where each heartbeat traces a three-dimensional loop in space. Figure 3 illustrates the typical morphology of a cardiac cycle projected onto the human thorax: the P-wave (atrial depolarization) appears as a small loop in pink, the QRS complex (ventricular depolarization) forms the dominant structure in blue, and the T-wave (ventricular repolarization) is shown in purple.

These deterministic coefficients represent a design choice prioritizing reproducibility and stability across diverse physiological conditions over subject-specific statistical optimization. While statistically derived coefficients (such as those by Feild et al. [21]) can achieve higher correlation with recorded ECGs on specific datasets (correlation coefficients > 0.9 across diverse patient populations), they rely on population-level regression and may exhibit reduced performance when individual anatomical variances deviate substantially from training data [17]. In contrast, the fixed transformation matrices *W* and *T* provide a stable, generalisable mapping derived from the dipole hypothesis and Frank’s lead-vector theory [15,20], ensuring consistent interpretation of the cardiac dipole vector irrespective of individual anatomical variances. This approach adheres to the principle of transparency in medical signal processing, where the relationship between raw surface potentials and reconstructed vector leads remains fixed and analytically traceable.

### 2.5. R-Peak Detection in the 3D Trajectory

From the transformed Frank-XYZ signals, $X\left(t\right)={\left(X\left(t\right),Y\left(t\right),Z\left(t\right)\right)}^{⊤}$, a three-dimensional kinematic signal is derived. Numerical first-order time derivatives (central differences), with time step dt defined as the mean sampling interval, yield the velocity-sum signal and its derivative as follows:

$$V_{\mathrm{sum}}\left(t\right)=\frac{dX}{dt}+\frac{dY}{dt}+\frac{dZ}{dt},\;\frac{dV_{\mathrm{sum}}}{dt}\left(t\right)=\frac{d^{2}X}{dt^{2}}+\frac{d^{2}Y}{dt^{2}}+\frac{d^{2}Z}{dt^{2}}.$$

**Figure 4:**
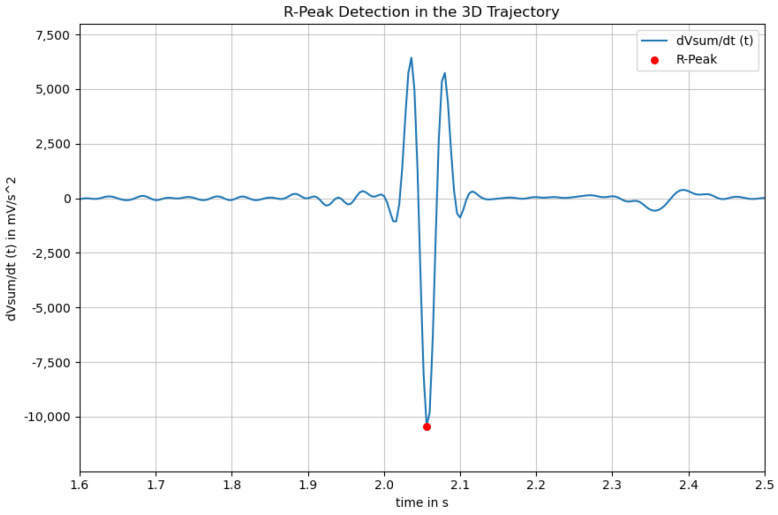
R-peak detection from the kinematic derivative signal $\frac{dV_{\mathrm{sum}}}{dt}\left(t\right)$, showing biphasic extrema (circles) corresponding to ventricular depolarization (QRS complex). Time in seconds.

**Figure 5:**
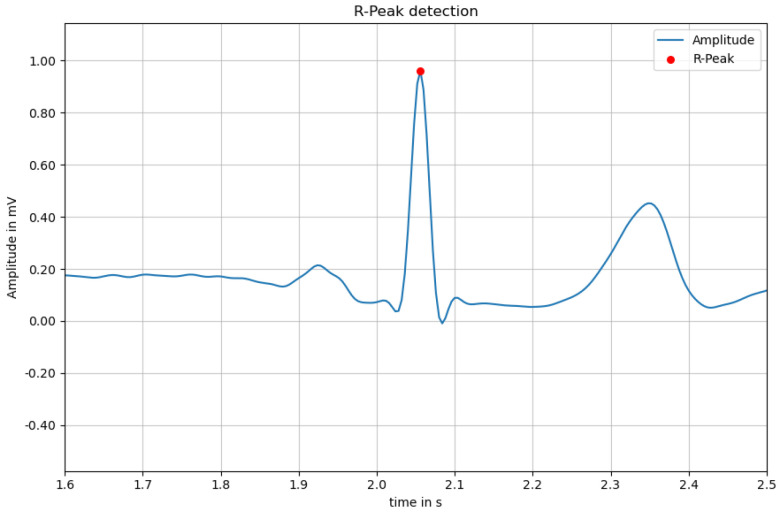
Detected R-peak visualized in a one-dimensional projection of the 3D VCG trajectory. Time in seconds.

This high-frequency kinematic signal $\frac{dV_{\mathrm{sum}}}{dt}\left(t\right)$ exhibits biphasic extrema (shown in Figure 4) in the QRS range and serves as a robust 3D basis for R-peak detection (shown in Figure 5).

Detection was performed directly on $\frac{dV_{\mathrm{sum}}}{dt}\left(t\right)$, which represents QRS activity as an energy-optimized aggregate. The algorithm combines local adaptive thresholding with a topological criterion and follows the general principle of the Pan–Tompkins algorithm [22], but is adapted to the 3D aggregate signal.

Adaptive normalization (median/IQR) replaces differentiation/squaring and detects R-peaks topologically (negative extremum between two maxima).

In contrast to the Pan–Tompkins algorithm, which is lead-specific and typically requires lead fusion, this approach operates on an isotropic 3D aggregate without multi-lead voting. It is independent of lead-specific differences in R-peak width or morphology, as it is based directly on 3D kinematics.

The resulting R-peak locations define the beat-to-beat time points for the HRV and Heartmovement analyses by enabling the estimation of the spatial cardiac dipole orientation per beat for EDR derivation.

### 2.6. Derivation of Beat-to-Beat Signals from the 3D Vectorcardiogram

At the detected R-peak time points, the corresponding Frank-XYZ coordinates $X_{R}={\left(X_{R},Y_{R},Z_{R}\right)}^{⊤}$ were extracted (starting from the second R-peak to enable interval-based comparisons). These discretized heart vector positions were converted to spherical coordinates as follows:

$$r_{R}=∥X_{R}∥,\;\theta_{R}=\mathrm{atan2}(Y_{R},X_{R}),\;\phi_{R}=\arccos\left(\frac{Z_{R}}{r_{R}+\varepsilon }\right).$$

The radial component $r_{R}$ describes the temporal amplitude modulation of the R-peak magnitude and corresponds to the classical one-dimensional EDR approach in which the R-peak amplitude of a single lead is used as a respiration surrogate [6].

The two orthogonal angular components $\phi_{R}$ and $\theta_{R}$ encode the spatial orientation of the cardiac dipole in two orthogonal directions. To combine both angles into a unified rotation surrogate, a complex signal was constructed as follows:

$$z_{R}=\phi_{R}+i\cdot \theta_{R}.$$

The derived EDR signal is defined as the magnitude of this complex signal at the discrete R-peak time points $t_{k}$, as follows:

$${\mathrm{EDR}}_{\mathrm{angular}}\left(t_{k}\right)=\left|z_{R}\left[k\right]\right|=\sqrt{\phi_{R}{\left[k\right]}^{2}+\theta_{R}{\left[k\right]}^{2}}.$$

**Figure 6:**
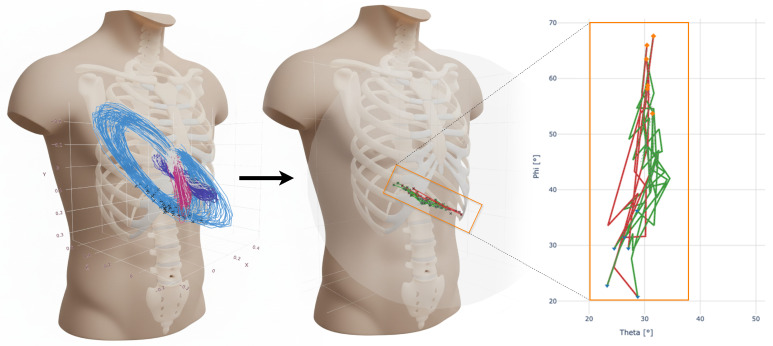
Derivation of beat-to-beat heart movement over a 60 s paced-breathing interval. (**Left**): 3D VCG loops with respiratory modulation, where the P loop is shown in pink, the QRS loop in blue, and the T loop in purple, colour-coded in the same way as in Figure 3. (**Center**): Detected R-peaks in the Frank-XYZ leads projected onto the unit sphere. (**Right**): Beat-to-beat rotation of the heart vector in $\phi_{R}$— and $\theta_{R}$- direction, color-coded for inspiration (red) and expiration (green).

**Figure 7:**
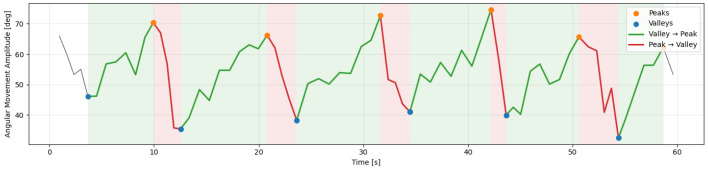
Beat-to-beat heart-movement signals over the same 60 s window as in Figure 6 during a 6 breaths per minute trial period. The angular Heartmovement signal is shown with inspiratory (red) and expiratory (green) phases highlighted.

This Euclidean norm serves as a robust one-dimensional surrogate that captures the total angular displacement of the cardiac axis. By combining both the azimuthal ($\theta_{R}$) and polar ($\phi_{R}$) variations, the metric becomes insensitive to subject-specific differences in the baseline anatomical heart axis and varying mechanical influences (e.g., chest versus abdominal breathing). It effectively tracks the global respiratory modulation (Figure 6 and Figure 7) without requiring complex spatial calibrations or computationally expensive 3D alignment steps.

### 2.7. RR Interval Signal

The third comparison signal, the RR-interval time series, was computed directly from the detected R-peak time points $t_{R}$ as follows:

$$\mathrm{RR-Interval}\left[k\right]=t_{R}\left[k+1\right]-t_{R}\left[k\right],\;k=1,2,\ldots ,N_{R}-1.$$

### 2.8. Signals Compared

To assess the ability to represent respiration frequency, three uniformly interpolated beat-to-beat signals were analyzed:

- ${\mathrm{RR-Interval}}_{\mathrm{EDR}}$: Frequency modulation of heart rate (RSA; autonomic influence);
- ${\mathrm{R-Amplitude}}_{\mathrm{EDR}}$: Amplitude modulation of R-peak magnitude (classical amplitude-based EDR surrogate);
- ${\mathrm{Heartmovement}}_{\mathrm{EDR}}$: Rotational amplitude of cardiac dipole (proposed 3D-EDR approach).

### 2.9. Adaptive Artifact Correction of Beat-to-Beat Signals

A multivariate artifact correction was implemented by extending the adaptive thresholding framework of Lipponen and Tarvainen [23], originally developed for univariate RR interval artifact detection, to a multivariate feature space. Instead of using the univariate ΔRR criterion, the present implementation computes the Mahalanobis distance [24,25] across five beat-to-beat features (${\mathrm{RR-Interval}}_{\mathrm{EDR}}$, ${\mathrm{R-Amplitude}}_{\mathrm{EDR}}$, ${\mathrm{Heartmovement}}_{\mathrm{EDR}}$, phase, and QRS area), as follows:

$$D_{M}\left(x_{k}\right)=\sqrt{{\left(x_{k}-\mu \right)}^{⊤}\Sigma^{-1}\left(x_{k}-\mu \right)}.$$

As a result, the same artifact mask can be applied consistently across all feature channels, supporting direct comparability between ${\mathrm{RR-Interval}}_{\mathrm{EDR}}$, ${\mathrm{R-Amplitude}}_{\mathrm{EDR}}$, and ${\mathrm{Heartmovement}}_{\mathrm{EDR}}$.

### 2.10. Reference Signal (Ground Truth)

According to the breathing exercise performed (described in Section 2.1), a reference signal was generated to evaluate the respiration estimates. For this purpose, a synthetic test signal was created to reproduce the temporal course of the guided breathing protocol used in the pilot study.

The reference signal was modeled as an asymmetric sawtooth waveform (shape parameter width=0.6) to obtain a physiologically plausible trajectory with a relatively slow rise during inhalation and faster decay during exhalation, as follows:

$${\mathrm{Ref}}_{\mathrm{signal}}\left(t\right)=A\cdot \mathrm{sawtooth}\left(2\pi f(t)\;t,\;\mathrm{width}=0.6\right),\;A=0.3.$$

f(t) represents the instantaneous breathing frequency, ranging from 0.1 Hz to 0.3 Hz.

The validity of this synthetic reference is supported by empirical evidence of adherence to acoustically paced breathing rhythms ranging from 8 to 18 breaths per minute in healthy test participants when compared with direct respiratory measures [26]. The resulting time series serves as the target signal for quantitatively evaluating the breathing frequency estimates derived from the three EDR candidates.

### 2.11. Spectral Processing and Quantitative Evaluation of the EDR Signals

The three artifact-corrected beat-to-beat signals (${\mathrm{RR-Interval}}_{\mathrm{EDR}}$, ${\mathrm{R-Amplitude}}_{\mathrm{EDR}}$, ${\mathrm{Heartmovement}}_{\mathrm{EDR}}$) were first interpolated to a common sampling rate of $F_{s}=10\;\mathrm{Hz}$. Instead of a high-pass filter with a corner frequency of 0.15 Hz that suppresses very slow respiratory components, a gentler high-pass at 0.05 Hz was used to preserve information on very slow breathing cycles (e.g., 6 bpm =0.1 Hz and below).

For each detrended signal, a scalogram was computed using a complex Morlet wavelet (B=2.5, C=1.5) [27,28] across 200 quasi-logarithmically spaced frequency scales in the range 0.01–0.445 Hz, as follows:

$$\mathrm{coeffs}\left(b,t\right)=\int y_{\det}\left(\tau \right)\;\psi_{b}^{*}\;\left(\frac{t-\tau }{b}\right)d\tau ,\;\mathrm{power}\left(b,t\right)={\left|\mathrm{coeffs}\left(b,t\right)\right|}^{2}.$$

**Figure 8:**
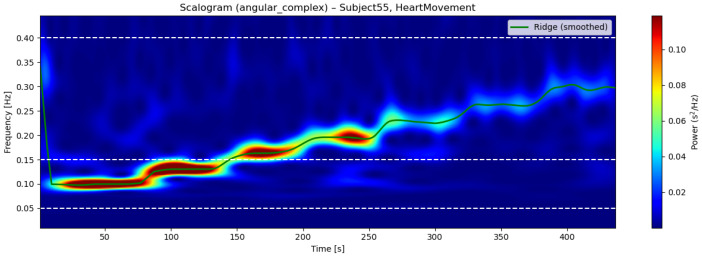
Scalogram of the ${\mathrm{Heartmovement}}_{\mathrm{EDR}}$ signal with the extracted CWT ridge (white dashed line) overlaid on the time–frequency power distribution.

The time-resolved dominant frequency (“ridge”) was determined as $\arg\max_{b}\;\mathrm{power}\left(b,t\right)$ at each time point and smoothed with a Savitzky–Golay filter (window 101, linear) to reduce noise-driven jitter. Compared to FFT-based peak methods, this continuous wavelet transform (CWT) ridge approach is more robust in terms of refSNR and is particularly suitable for non-stationary breathing frequency sweeps [28,29]. An exemplary scalogram of the ${\mathrm{Heartmovement}}_{\mathrm{EDR}}$ signal with the extracted ridge is shown in Figure 8.

Because the exact start of the guided breathing protocol relative to the ECG recording was unknown, the z-normalized EDR signals were aligned to the synthetic reference signal using cross-correlation as follows:

$$r\left(\tau \right)=\frac{1}{N}\sum_{t}y_{\mathrm{EDR}}(t)\;s_{\mathrm{test}}(t+\tau ).$$

**Figure 9:**
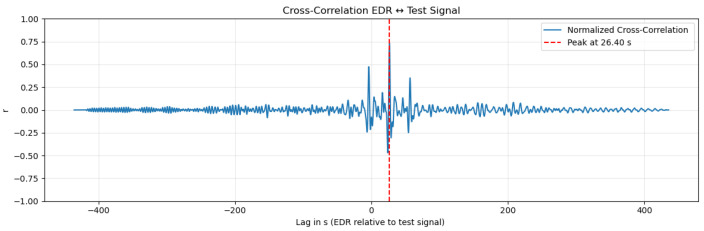
Representative cross-correlation between the z-normalized ${\mathrm{Heartmovement}}_{\mathrm{EDR}}$ signal and the synthetic reference signal. The peak indicates the synchronization lag τ used to align both signals prior to evaluation.

The lag τ with maximum correlation (typically 5–20 s) was used for synchronization; both signals were then trimmed such that only the overlapping time interval was retained for subsequent analysis (see Figure 9 for a representative cross-correlation result).

Alignment lags were inspected in a representative subset of recordings and found to be consistent across EDR methods within the same participant, with differences below one respiratory cycle at the highest tested breathing rate (≤3 s at 18 bpm). Segments in which alignment failed would produce a low refSNR and are therefore captured by the quality-control procedures described in Section 3.1.

**Figure 10:**
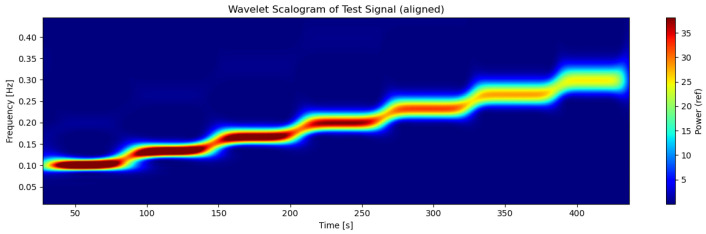
Scalogram of the synthetic reference signal. The respiration band (0.05–0.50 Hz) used for mask construction is bounded by the dashed horizontal lines.

Within the respiration band (0.05–0.50 Hz) of the reference scalogram (Figure 10), a nonlinear soft mask was defined as follows:

$$\mathrm{mask}\left(b,t\right)=\mathrm{clip}\;\left(1.5\cdot {\left(\frac{P_{\mathrm{ref}}\left(b,t\right)}{\max P_{\mathrm{ref}}}\right)}^{0.2},\;0,\;1\right).$$

Multiplying this mask with the EDR scalogram separates respiration-dominated components

$$P_{\mathrm{sig}}\left(b,t\right)=P_{\mathrm{EDR}}\left(b,t\right)\cdot \mathrm{mask}\left(b,t\right)$$

from residual/noise components

$$P_{\mathrm{noise}}\left(b,t\right)=P_{\mathrm{EDR}}\left(b,t\right)\cdot \left(1-\mathrm{mask}(b,t)\right).$$

**Figure 11:**
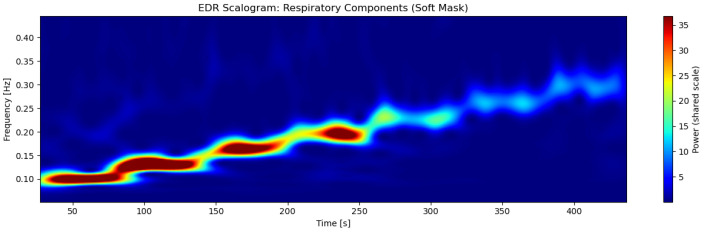
Scalogram of the respiration-dominated component $P_{\mathrm{sig}}$ of the ${\mathrm{Heartmovement}}_{\mathrm{EDR}}$ signal after applying the soft mask from one test participant.

**Figure 12:**
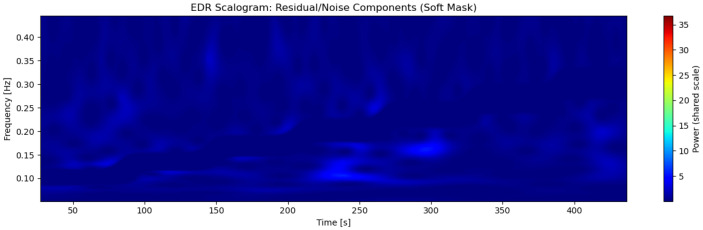
Scalogram of the residual/noise component $P_{\mathrm{noise}}$ of the ${\mathrm{Heartmovement}}_{\mathrm{EDR}}$ signal after applying the inverted soft mask from one test participant.

The resulting separation is illustrated in Figure 11 and Figure 12.

This reference-derived mask is methodologically justified under the controlled paced-breathing protocol used in this study: participants were shown to adhere closely to the instructed breathing frequencies [26], such that the dominant respiratory spectral energy is expected at the known target-frequency trajectory. Spectral power outside this trajectory can therefore legitimately be treated as non-respiratory noise. Importantly, because the identical mask is applied to all three EDR methods, the relative comparison between methods remains unbiased.

Both components are defined as the frequency-integrated squared wavelet coefficients ($\sum_{b}{\left|W\right|}^{2}$) within the respiratory band (0.05–0.50 Hz), weighted by the soft time–frequency mask and its complement, respectively. The time-resolved signal-to-noise ratio was then defined as

$$\mathrm{refSNR}(t)=10\log_{10}\left(\frac{\sum_{b}P_{\mathrm{sig}}\left(b,t\right)}{\sum_{b}P_{\mathrm{noise}}\left(b,t\right)}\right).$$

The 60 s segments corresponding to the target breathing frequencies (6–18 bpm) were identified automatically. For each segment, the following key metrics were computed:

- Reference-based signal-to-noise ratio (refSNR): Median refSNR within the respiration band, expressed in dB, quantifying how strongly the EDR signal concentrates spectral power around the externally prescribed target breathing frequency relative to non-respiratory spectral components. Under the paced-breathing protocol, the reference trajectory is treated as the target signal for this analysis, enabling a robust and method-comparable refSNR estimate.
- Estimation error: Median signed deviation of the CWT ridge frequency from the target frequency in Hz ($\varepsilon ={\hat{f}}_{\mathrm{resp}}-f_{\mathrm{resp}}$).

Classical 1D-EDR approaches are known to exhibit increased estimation errors, particularly at lower breathing frequencies, due to spectral overlap between respiratory and baroreceptor components [6]. By combining CWT-based ridge tracking instead of simple FFT peaks, multivariate Mahalanobis-based artifact correction, preservation of very low-frequency components via gentle detrending, and explicit use of 3D rotational information, the proposed pipeline specifically addresses these limitations. The derived segment-wise metrics—refSNR and estimation error, along with HRV parameters—were exported to CSV files for each participant and EDR method, enabling aggregation across participants and a quantitative comparison of the three respiration surrogates (${\mathrm{RR-Interval}}_{\mathrm{EDR}}$, ${\mathrm{R-Amplitude}}_{\mathrm{EDR}}$, ${\mathrm{Heartmovement}}_{\mathrm{EDR}}$).

### 2.12. Statistical Analysis

To assess the effects of the EDR method and breathing rate on estimation accuracy, we fitted linear mixed-effects models (LMMs) using the MixedLM function from the Python statsmodels library [30]. The dependent variable was the signed frequency estimation error (EDR-estimated minus target frequency, in Hz). EDR method (three levels: ${\mathrm{RR-Interval}}_{\mathrm{EDR}}$, ${\mathrm{R-Amplitude}}_{\mathrm{EDR}}$, ${\mathrm{Heartmovement}}_{\mathrm{EDR}}$; reference: ${\mathrm{Heartmovement}}_{\mathrm{EDR}}$) and target breathing rates (seven levels) were included as fixed effects, along with their two-way interaction. Mean RR interval was additionally included as a continuous covariate to examine whether estimation accuracy is influenced by heart rate and, if so, whether it differs between EDR methods. Subject identifier was modeled as a random intercept (1∣Subject) to account for the repeated-measures structure of the data. All continuous predictors were *z*-standardized prior to model fitting. Models were estimated using restricted maximum likelihood (REML); fixed-effect significance was assessed via Wald $\chi^{2}$ tests (α=0.05); and model selection was guided by AIC and BIC.

To assess potential multicollinearity between continuous predictors, variance inflation factors (VIFs) were computed prior to model fitting. Both bpm_target and hrv_mean_rr_ms yielded VIF=1.04 (Pearson r=−0.19), indicating negligible collinearity [31]. Coefficient stability was confirmed by comparing models with and without covariates; method contrasts changed by less than 12% and remained significant in all cases [32]. To further isolate the independent contribution of the mean RR interval on estimation accuracy while avoiding sequential over-adjustment under respiratory sinus arrhythmia, partial correlations in Section 3.4 were derived from a single multivariable OLS model [33].

Furthermore, systematic bias for each EDR method was assessed using one-sample *t*-tests against the target frequency. As error distributions violated normality (Shapiro–Wilks test), overall between-method differences in estimation accuracy were evaluated with the Kruskal–Wallis test, followed by pairwise post hoc comparisons using Dunn’s test with Bonferroni correction. All analysis code is publicly available as open source.

As a final step of evaluation, the estimation error of ${\mathrm{Heartmovement}}_{\mathrm{EDR}}$ was compared with the EDR estimates determined by the Kubios-HRV Premium software package (version 3.5.0) [8], which represents the most widely applied HRV-analysis software. The EDR algorithm implemented in Kubios-HRV combines R-peak amplitude modulation and RR interval modulation; the exact algorithmic weighting of both signal sources is proprietary and not publicly disclosed. The left midaxillary lead (referenced against manubrium sterni) was selected because it is equivalent to the bipolar Einthoven Lead II, which represents the largest R-peak amplitude for most anatomical heart location types. The automatic artifact correction of Kubios-HRV was applied, and nine segments out of 133 in which the proportion of corrected beats exceeded 5% were discarded; the remaining 124 segments were drawn from the same 19 participants as the primary analysis, with partial overlap in excluded segments between the two pipelines. As preprocessing differs between the present pipeline and Kubios-HRV, the Kubios-EDR values were not entered into the LMM analysis; instead, indications of significant differences were tested by two-way ANOVA (2 EDR methods and 7 breathing rates) and post hoc Holm-corrected paired *t*-tests.

## 3. Results

The following results compare the performance of ${\mathrm{RR-Interval}}_{\mathrm{EDR}}$, ${\mathrm{R-Amplitude}}_{\mathrm{EDR}}$, and ${\mathrm{Heartmovement}}_{\mathrm{EDR}}$ across all target breathing frequencies. Key metrics, including reference-based signal-to-noise ratio (refSNR) and frequency estimation error, were derived for each participant, as described in Section 2.10 and Section 2.11.

### 3.1. Aggregated Evaluation and Quality Control

The segment-wise quality-control (QC) files from all participants and sessions were merged into a single dataset. Prior to analysis, a refSNR-based subject selection was applied: for each participant, the median refSNR was computed across all three EDR methods (${\mathrm{RR-Interval}}_{\mathrm{EDR}}$, ${\mathrm{R-Amplitude}}_{\mathrm{EDR}}$, and ${\mathrm{Heartmovement}}_{\mathrm{EDR}}$), and only participants with a median refSNR of at least 1.0 dB were included in the analysis.

**Figure 13:**
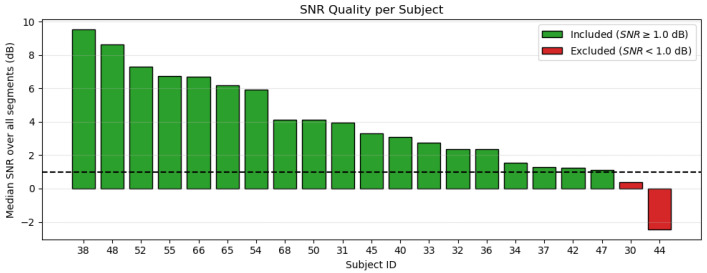
Median refSNR per participant (red: below threshold; green: above threshold).

This procedure excluded two recordings, leaving N=19 participants for subsequent analysis (Figure 13). Exclusion was based on the previously defined participant-level refSNR criterion: participants were retained only if the median refSNR across ${\mathrm{RR-Interval}}_{\mathrm{EDR}}$, ${\mathrm{R-Amplitude}}_{\mathrm{EDR}}$, and ${\mathrm{Heartmovement}}_{\mathrm{EDR}}$ was at least 1.0 dB. The two excluded recordings did not meet this threshold.

Post hoc inspection of the two excluded recordings revealed severe signal artifacts causing R-peak detection failures, resulting in implausible interbeat intervals exceeding 4 s across all three EDR methods, confirming that these data were unsuitable for analysis.

### 3.2. Signal-to-Noise Ratio and Frequency Robustness

Throughout the following, refSNR refers to the reference-based signal-to-noise ratio defined in Section 2.11, quantifying spectral alignment with the paced-breathing trajectory rather than conventional broadband SNR.

**Figure 14:**
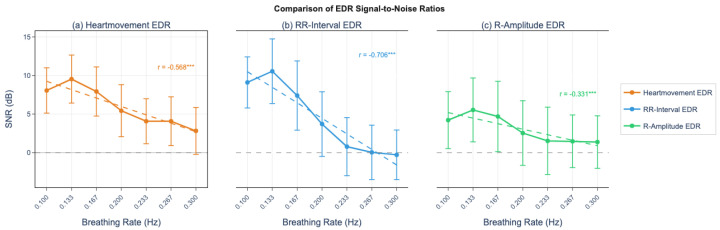
Mean refSNR (±1 SD) per method as a function of breathing frequency. The dashed line marks refSNR =0 dB. ${\mathrm{RR-Interval}}_{\mathrm{EDR}}$ drops below the noise floor above 12 bpm; ${\mathrm{Heartmovement}}_{\mathrm{EDR}}$ remains positive across the entire range. *N* decreases at 18 bpm due to segment exclusion (HM: *N* = 18; PR: *N* = 16; RA: *N* = 13). *** *p* < 0.001.

Across all breathing rates, ${\mathrm{Heartmovement}}_{\mathrm{EDR}}$ achieved the highest mean refSNR (6.01±3.83 dB), followed by ${\mathrm{RR-Interval}}_{\mathrm{EDR}}$ (4.62±5.64 dB) and ${\mathrm{R-Amplitude}}_{\mathrm{EDR}}$ (3.20±4.24 dB). The methods diverged markedly with increasing breathing rate (Figure 14): ${\mathrm{RR-Interval}}_{\mathrm{EDR}}$ exhibits the steepest frequency-dependent collapse (r=−0.706, p<0.001), dropping below the noise floor above 12 bpm (−0.28±3.21 dB at 18 bpm), while ${\mathrm{Heartmovement}}_{\mathrm{EDR}}$ maintains a positive refSNR across the full frequency range (r=−0.568, p<0.001; 2.82±3.04 dB at 18 bpm).

### 3.3. Estimation Accuracy

**Figure 15:**
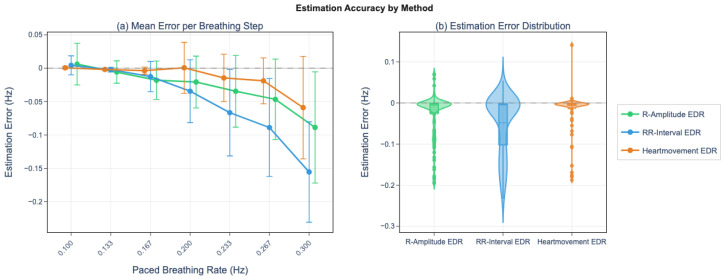
Estimation accuracy across EDR methods (*N* = 380, 19 subjects). (**a**) Mean signed estimation error ± SD per paced breathing rate step, illustrating the frequency-dependent accuracy loss; the steeper decline of ${\mathrm{RR-Interval}}_{\mathrm{EDR}}$ reflects the interaction term identified by the LMM ($\hat{\beta }=-0.034$ Hz/SD, p<0.001). (**b**) Distribution of signed estimation error across all segments; boxes indicate IQR and median, violin shows the full empirical distribution. Reference category: ${\mathrm{Heartmovement}}_{\mathrm{EDR}}$; dashed line denotes zero error.

All three methods exhibited a systematic negative bias (all p<0.001, one-sample *t*-test), indicating a general tendency to underestimate the target respiratory frequency. The magnitude of this bias differed substantially: ${\mathrm{Heartmovement}}_{\mathrm{EDR}}$ achieved a mean bias of only −0.014±0.041 Hz (−0.81±2.46 br/min; MAE =0.016 Hz, RMSE =0.043 Hz), compared to −0.048±0.071 Hz (−2.89±4.26 br/min; MAE =0.050 Hz, RMSE =0.086 Hz) for ${\mathrm{RR-Interval}}_{\mathrm{EDR}}$ and −0.026±0.053 Hz (−1.58±3.18 br/min; MAE =0.032 Hz, RMSE =0.059 Hz) for ${\mathrm{R-Amplitude}}_{\mathrm{EDR}}$. The full error distributions are visualised in panel (b) of Figure 15.

The LMM confirmed a significant main effect of the EDR method ($\chi^{2}\left(2\right)=41.2$, p<0.001). Post hoc Welch’s *t*-tests showed that ${\mathrm{Heartmovement}}_{\mathrm{EDR}}$ significantly outperformed both ${\mathrm{RR-Interval}}_{\mathrm{EDR}}$ ($t_{204}=4.81$, $\Delta \hat{\mu }=+0.035$ Hz, p<0.001) and ${\mathrm{R-Amplitude}}_{\mathrm{EDR}}$ ($t_{222}=2.13$, $\Delta \hat{\mu }=+0.013$ Hz, p=0.035). ${\mathrm{R-Amplitude}}_{\mathrm{EDR}}$ in turn outperformed ${\mathrm{RR-Interval}}_{\mathrm{EDR}}$ ($t_{236}=-2.76$, p=0.006), establishing a clear accuracy ranking: Heartmovement > R-Amplitude > RR-Interval.

Beyond the overall method difference, all three methods showed a pronounced frequency-dependent accuracy loss: estimation error increased systematically with breathing rate for all methods ($\chi^{2}\left(1\right)=18.8$, p<0.001; Pearson r=−0.383, −0.689, −0.487 for ${\mathrm{Heartmovement}}_{\mathrm{EDR}}$, $\mathrm{RR}-{\mathrm{Interval}}_{\mathrm{EDR}}$, and ${\mathrm{R-Amplitude}}_{\mathrm{EDR}}$, respectively; all p<0.001). Critically, this deterioration interacted significantly with the EDR method ($\chi^{2}\left(2\right)=39.6$, p<0.001), and was most severe for ${\mathrm{RR-Interval}}_{\mathrm{EDR}}$ (LMM interaction $\hat{\beta }=-0.034$ Hz/SD, z=−6.21, p<0.001), approximately twice the rate of decline observed for ${\mathrm{Heartmovement}}_{\mathrm{EDR}}$ (${\hat{\beta }}_{\mathrm{base}}=-0.017$ Hz/SD, z=−4.34, p<0.001). The interaction term for ${\mathrm{R-Amplitude}}_{\mathrm{EDR}}$ was also significant but less pronounced ($\hat{\beta }=-0.012$ Hz/SD, z=−2.05, p=0.040). This frequency-dependent accuracy loss is illustrated in panel (a) of Figure 15.

Finally, the mean RR interval exerted a significant independent effect on estimation error beyond breathing rate ($\chi^{2}\left(1\right)=5.91$, p=0.015; $\hat{\beta }=-0.008$ Hz/SD, z=−2.43, p=0.015). This relationship is examined in detail in Section 3.4.

### 3.4. Heart Period Effects on Estimation Accuracy

**Figure 16:**
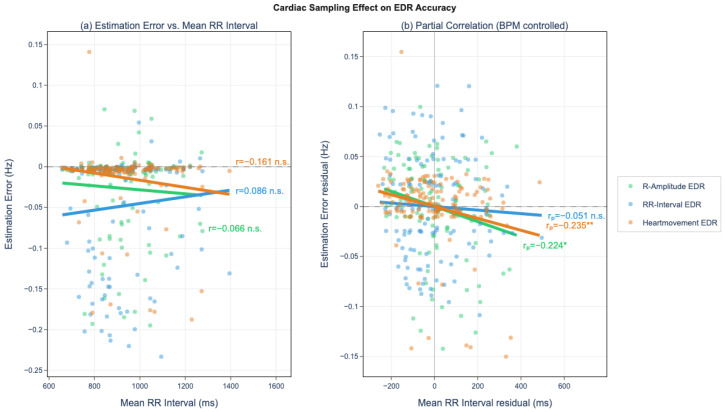
Associations between mean RR interval and estimation accuracy. (**a**) Raw (non-residualized) estimation error vs. mean RR interval; none of the correlations reach significance (p>0.05), as breathing rate and heart rate co-vary across subjects. (**b**) Partial correlation between mean RR interval and estimation error, controlling for breathing rate via a single multivariable OLS model; significant negative partial correlations indicate that longer RR intervals—fewer cardiac cycles per window—increase estimation error independently of breathing rate for the two chest movement-related methods. Scatter plots with OLS regression lines; partial Pearson *r* and significance level annotated per method. Reference: ${\mathrm{Heartmovement}}_{\mathrm{EDR}}$. ** *p* < 0.01; * *p* < 0.05; ns *p* ≥ 0.05.

To further evaluate the source of this effect and whether it differed across EDR methods, we computed ordinary least squares (OLS) regressions of the estimation error over the mean RR interval separately for the three EDR methods. To isolate the independent contribution of heart period duration while controlling for breathing rate, we fitted a single multivariable OLS regression ($\hat{\varepsilon }∼\mathrm{BMP}\_\mathrm{target}+\mathrm{hrv}\_\mathrm{mean}\_\mathrm{rr}\_\mathrm{ms}$) per EDR method. Partial correlations between mean RR interval and estimation error, controlling for breathing rate, were derived from the same model. The variance inflating factor was below 1.08 across all methods, confirming negligible collinearity between predictors [31]. Both analyses are shown in Figure 16.

Panel (a) of Figure 16 shows the raw associations between the mean RR interval and estimation error. None of the three methods yielded a significant correlation in the raw analysis (${\mathrm{Heartmovement}}_{\mathrm{EDR}}$: r=−0.161, p=0.065; ${\mathrm{RR-Interval}}_{\mathrm{EDR}}$: r=+0.086, p=0.331; ${\mathrm{R-Amplitude}}_{\mathrm{EDR}}$: r=−0.066, p=0.479), which is expected given that breathing rate and heart rate co-vary across subjects and conditions, masking the independent contribution of heart period duration.

Panel (b) shows the partial correlations between the mean RR interval and estimation error after controlling for breathing rate within a single multivariable model. Here, a dissociation between the two signal classes becomes apparent. The two chest movement-related methods both showed significant negative partial correlations: ${\mathrm{Heartmovement}}_{\mathrm{EDR}}$ ($r_{\mathrm{partial}}=-0.235$, p=0.007, OLS slope =−0.017 Hz/ms) and ${\mathrm{R-Amplitude}}_{\mathrm{EDR}}$ ($r_{\mathrm{partial}}=-0.224$, p=0.015, OLS slope =−0.029 Hz/ms). This indicates that longer RR intervals are associated with larger estimation errors independently of breathing rate. In contrast, ${\mathrm{RR-Interval}}_{\mathrm{EDR}}$ showed no significant partial correlation ($r_{\mathrm{partial}}=-0.051$, p=0.571).

### 3.5. Summary and Method Ranking

The key evaluation metrics are summarized in Table 1. Across all dimensions, ${\mathrm{Heartmovement}}_{\mathrm{EDR}}$ achieved the best overall performance: lowest estimation bias (MAE =0.016 Hz), highest mean refSNR (6.01 dB), most moderate frequency-dependent decline, and a significant heart period duration effect consistent with chest movement-related signal generation (r=−0.233, p=0.007; Section 3.4).

${\mathrm{RR-Interval}}_{\mathrm{EDR}}$ is competitive at low breathing rates (6–10 bpm), where RSA amplitude is large, but its refSNR collapses below the noise floor above 12 bpm and its estimation error increases at approximately twice the rate of ${\mathrm{Heartmovement}}_{\mathrm{EDR}}$ (LMM interaction $\hat{\beta }=-0.034$ Hz/SD, p<0.001). Crucially, this degradation cannot be compensated by a higher heart period duration, as evidenced by the absent residual correlation in Section 3.4, pointing to neurovegetative confounding as a fundamental signal-level limitation.

### 3.6. Comparison with Kubios-HRV Estimate of EDR

Finally, estimation accuracy of ${\mathrm{Heartmovement}}_{\mathrm{EDR}}$ was compared against the EDR results using Kubios-HRV (version 3.5.0, [8]).

A two-way ANOVA with subject as a blocking factor revealed a significant main effect of method (F(1,224)=44.55, p<0.001, $\eta_{p}^{2}=0.166$), a significant main effect of breathing rate (F(6,224)=12.46, p<0.001, $\eta_{p}^{2}=0.250$), and a significant method × breathing rate interaction (F(6,224)=4.31, p<0.001, $\eta_{p}^{2}=0.104$), indicating that the relative performance of the two methods was not uniform across the tested breathing rates.

**Figure 17:**
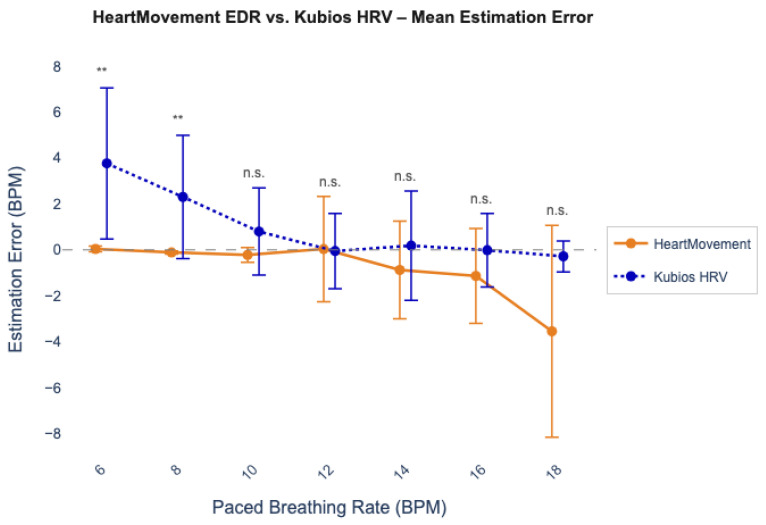
Mean estimation error (±95% CI) per method and paced breathing rate for ${\mathrm{Heartmovement}}_{\mathrm{EDR}}$ and Kubios-HRV. Positive values indicate overestimation of the target breathing rate. Significance annotations reflect Holm-corrected pairwise contrasts (Kubios–${\mathrm{Heartmovement}}_{\mathrm{EDR}}$; ** *p* < 0.01; n.s. *p* ≥ 0.05).

Post hoc pairwise contrasts (paired *t*-tests, Holm-corrected) suggest that this interaction was driven by systematic divergence at the lower breathing rates (see Table 2 and Figure 17). At 6 BPM, Kubios-HRV overestimated the respiratory rate by Δ=+3.72 BPM relative to ${\mathrm{HeartMovement}}_{\mathrm{EDR}}$ (t(17)=−4.82, $p_{\mathrm{Holm}}=0.001$), and the bias remained significant at 8 BPM (Δ=+2.42 BPM, t(18)=−3.91, $p_{\mathrm{Holm}}=0.006$). At 10 BPM, the raw contrast was significant (p=0.039) but did not survive Holm correction ($p_{\mathrm{Holm}}=0.157$, Δ=+1.02 BPM). No significant difference was observed at 12, 14, or 16 BPM (all $p_{\mathrm{Holm}}>0.05$). At slow breathing rates (6–8 BPM), Kubios-HRV showed a systematic positive bias relative to ${\mathrm{HeartMovement}}_{\mathrm{EDR}}$.

## 4. Discussion

The present work shows that exploiting three-dimensional cardiac vector information via ${\mathrm{Heartmovement}}_{\mathrm{EDR}}$ enables substantially more robust breathing frequency estimation than conventional single-lead EDR approaches. At breathing rates between 6 and 10 bpm, interindividual variance is impressively low for ${\mathrm{Heartmovement}}_{\mathrm{EDR}}$ when compared with ${\mathrm{R-Amplitude}}_{\mathrm{EDR}}$ and ${\mathrm{RR-Interval}}_{\mathrm{EDR}}$. In contrast, the EDR algorithm implemented in the Kubios-HRV software [8] yields high precision at elevated breathing rates, but performs substantially worse at slow breathing rates. The superiority of ${\mathrm{Heartmovement}}_{\mathrm{EDR}}$ at breathing rates between 6 and 10 bpm is particularly relevant for applications where precise respiration tracking is essential for interpreting vagally mediated HRV components [9,34], such as during relaxation techniques, resonance breathing or biofeedback protocols.

A further finding reveals that higher cardiac sampling density at increased heart rates was associated with improved EDR accuracy for methods based on chest movement, consistent with the interpretation that more cardiac cycles per estimation window improve spectral resolution of the respiratory frequency. In contrast, ${\mathrm{RR-Interval}}_{\mathrm{EDR}}$ is fundamentally limited by the inverse relationship between heart rate and RSA [35]. The higher the heart rate, the lower is vagally mediated RSA, rendering interval-based breathing-frequency estimation less precise.

From a physiological perspective, the advantage of the ${\mathrm{Heartmovement}}_{\mathrm{EDR}}$ signal can be explained by its direct representation of respiration-related rotation and translation of the cardiac dipole in three-dimensional space driven by thoracic motion during inhalation and exhalation [10,11]. In contrast, ${\mathrm{RR-Interval}}_{\mathrm{EDR}}$ primarily reflects the RSA effect, which is prone to the complexity of autonomic cardiorespiratory interaction [36]. ${\mathrm{R-Amplitude}}_{\mathrm{EDR}}$, in turn, represents only a one-dimensional projection of the underlying 3D motion. The higher correlation with the target breathing frequency improved refSNR and reduced frequency-dependent degradation, supporting the interpretation that 3D rotational information constitutes a physiologically closer surrogate marker of respiration than commonly used EDR metrics.

The comparison with EDR estimates using Kubios-HRV raises the question of whether differences in preprocessing filter design contribute to the observed performance divergence across breathing rates. ${\mathrm{Heartmovement}}_{\mathrm{EDR}}$, by contrast, maintained near-zero bias across the slow breathing range, reflecting the lower 0.05 Hz cutoff chosen to preserve the full biomechanical bandwidth. The non-significant reversal at 18 BPM is nonetheless consistent with the hypothesis that a more restrictive high-pass filter suppresses low-frequency spectral leakage at elevated breathing rates, where baseline wander or sympathetic LF oscillations may contaminate the EDR spectrum [6].

Methodologically, the proposed pipeline addresses several known limitations of established EDR procedures [6]: Avoiding narrow bandpass filters in the classical respiration band prevents the loss of very slow breathing patterns around 6 bpm (0.1 Hz), as encountered during resonance-frequency breathing and vagus-activating breathing exercises. Furthermore, our multivariate artifact correction based on Mahalanobis distance leverages redundancy between HRV variability, amplitude modulation, and rotation parameters to detect outliers more robustly than purely univariate thresholding. Wavelet-based ridge analysis provides a practical trade-off between frequency and time resolution and thereby supports consistent handling of non-stationary breathing frequency sweeps, frequently used in training and experimental protocols [27,28].

While the controlled within-subject design enabled comparison under identical signal-processing conditions, several boundary conditions constrain the generalizability of these findings. This study was conducted on healthy participants using idealized, paced breathing and a specific mobile EASI system. Generalizability to clinical populations, unguided spontaneous breathing, or other electrode configurations (e.g., standard 12-lead ECG) should be examined in future work. Moreover, only low-to-moderate breathing frequencies (6–18 bpm) were investigated; very high frequencies and strongly irregular breathing patterns were outside the scope of this analysis. Furthermore, the influence of cardiac sampling density on EDR accuracy—while statistically significant—should be interpreted with caution, as mean RR interval may also reflect fitness level, autonomic tone, age, or other subject-level factors not controlled in the present study. A prospective design with systematically varied heart rates, e.g., during physical exercises, would be needed to establish this effect more rigorously.

Building on the present findings, further validation against spirometry data appears promising. Direct spirometric measurements were not available in the present study. However, a study by Ritz and Dahme [26] demonstrates that healthy adults are able to adhere closely to paced breathing protocols. The authors report mean deviations of +0.3, −0.1, −0.3, and −0.9 breaths per minute from direct respiration measurements at paced rates of 8, 10.5, 13, and 18 breaths per minute, respectively. Therefore, the degree of deviation of all three EDR methods from the target breathing frequency may be in fact smaller, but it would not change the observed differences across EDR methods. Although our results demonstrate the superiority of the 3D heart movement approach in comparison to other established EDR methods, the relatively small number of only healthy participants limit a recommendation for use in the variety of clinical populations. This feasibility study was primarily meant to address the present shortcomings of existing EDR methods used for HRV analysis.

## 5. Conclusions

The results of our study demonstrate the feasibility of the EASI-based 3D-EDR approach for breathing frequency estimation under controlled conditions, and suggest particular promise at slow breathing rates below 0.15 Hz, where conventional single-lead EDR methods are known to fail. Consequently, the estimation of respiratory HRV may benefit from the proposed 3D approach, especially when vagal activation procedures are examined. Future work should validate the approach against direct spirometric measurements and extend it to spontaneous breathing conditions in clinical populations. 

## Figures and Tables

**Table 1:** Key evaluation metrics for the three EDR methods (best value per row in **bold**).

Metric	${\mathrm{Heartmovement}}_{\mathrm{EDR}}$	${\mathrm{RR-Interval}}_{\mathrm{EDR}}$	${\mathrm{R-Amplitude}}_{\mathrm{EDR}}$
Mean refSNR (dB)	**6.01**	4.62	3.20
refSNR @ 18 bpm (dB)	**2.82**	−0.28	1.38
Mean Bias (Hz)	−0.014	−0.048	−0.026
Mean Bias (Bpm)	−0.84	−2.88	−1.56
MAE (Hz)	**0.016**	0.050	0.032
RMSE (Hz)	**0.043**	0.086	0.059
*r* (Residual | Mean RR)	−**0.235** **	$-0.051^{\;n.s.}$	−0.224 *

** *p* < 0.01; * *p* < 0.05; n.s. *p* ≥ 0.05.

**Table 2:** Pairwise contrasts (Kubios–${\mathrm{Heartmovement}}_{\mathrm{EDR}}$) at each paced breathing rate. Positive Δ indicates Kubios overestimates relative to ${\mathrm{Heartmovement}}_{\mathrm{EDR}}$. *p*-values are Holm-corrected across seven comparisons (α=0.05). *** *p* < 0.001; ** *p* < 0.01; * *p* < 0.05; n.s. *p* ≥ 0.05.

BPM	*n*	${\bar{e}}_{\mathrm{HM}}$	${\bar{e}}_{\mathrm{Ku}}$	Δ	*t*	$p_{\mathrm{raw}}$	Significance	$p_{\mathrm{Holm}}$	Significance
6	18	+0.04	+3.76	+3.72	−4.82	<0.001	***	0.001	**
8	19	−0.11	+2.30	+2.42	−3.91	0.001	**	0.006	**
10	19	−0.22	+0.80	+1.02	−2.22	0.039	*	0.157	n.s.
12	17	+0.32	−0.05	−0.37	+0.59	0.563	n.s.	0.652	n.s.
14	16	−0.44	+0.18	+0.62	−1.02	0.326	n.s.	0.652	n.s.
16	17	−1.23	−0.01	+1.22	−1.80	0.090	n.s.	0.270	n.s.
18	17	−3.61	−0.29	+3.32	−2.63	0.018	*	0.092	n.s.

## Data Availability

The raw ECG and respiratory data, as well as all processed datasets, are openly available at the project’s GitHub 3.5.3 repository. The complete analysis pipeline, including the computational environment and Jupyter notebooks required to replicate the reported results, is accessible under the CC BY-NC-SA 4.0 license at https://github.com/FelixKuon/BeyondSingleLeadEDR (accessed on 2 June 2026).

## Abbreviations

The following abbreviations are used in this manuscript:

AIC	Akaike Information Criterion
BIC	Bayesian Information Criterion
bpm	Breaths per minute
CWT	Continuous Wavelet Transform
EASI	Einthoven, Augmented, Spine, Inferior (electrode system)
ECG	Electrocardiogram
EDR	Electrocardiogram-Derived Respiration
FIR	Finite Impulse Response
HF	High Frequency
HRV	Heart Rate Variability
LF	Low Frequency
LF/HF	Low Frequency/High Frequency ratio
LMM	Linear Mixed-Effects Model
MAE	Mean Absolute Error
OLS	Ordinary Least Squares
OSA	Obstructive Sleep Apnea
QC	Quality Control
REML	Restricted Maximum Likelihood
RMSE	Root Mean Square Error
RSA	Respiratory Sinus Arrhythmia
SD	Standard Deviation
refSNR	Reference-based Signal-to-Noise Ratio
VCG	Vectorcardiogram
